# Genetic Population Structure of *Tectura paleacea*: Implications for the Mechanisms Regulating Population Structure in Patchy Coastal Habitats

**DOI:** 10.1371/journal.pone.0018408

**Published:** 2011-04-07

**Authors:** Emina Begovic, David R. Lindberg

**Affiliations:** 1 Department of Molecular and Cell Biology, University of California, Berkeley, California, United States of America; 2 Department of Integrative Biology and Museum of Paleontology, University of California, Berkeley, California, United States of America; California Academy of Sciences, United States of America

## Abstract

The seagrass limpet *Tectura paleacea* (Gastropoda; Patellogastropoda) belongs to a seagrass obligate lineage that has shifted from the Caribbean in the late Miocene, across the Isthmus of Panama prior to the closing of the Panamanian seaway, and then northward to its modern Baja California – Oregon distribution. To address whether larval entrainment by seagrass beds contributes to population structuring, populations were sampled at six California/Oregon localities approximately 2 degrees latitude apart during two post-settlement periods in July 2002 and June 2003. Partial cytochrome oxidase b (Cytb) sequences were obtained from 20 individuals (10 per year) from each population in order to determine the levels of population subdivision/connectivity. From the 120 individuals sequenced, there were eighty-one unique haplotypes, with the greatest haplotype diversity occurring in southern populations. The only significant genetic break detected was consistent with a peri-Point Conception (PPC) biogeographic boundary while populations north and south of Point Conception were each panmictic. The data further indicate that populations found south of the PPC biogeographic boundary originated from northern populations. This pattern of population structure suggests that seagrass patches are not entraining the larvae of *T. paleacea* by altering flow regimes within their environment; a process hypothesized to produce extensive genetic subdivision on fine geographic scales. In contrast to the haplotype data, morphological patterns vary significantly over very fine geographic scales that are inconsistent with the observed patterns of genetic population structure, indicating that morphological variation in *T. paleacea* might be attributed to differential ecophenotypic expression in response to local habitat variability throughout its distribution. These results suggest that highly localized conservation efforts may not be as effective as large-scale conservation efforts in near shore marine environments.

## Introduction

The effects of larval dispersal on marine invertebrate ecology and evolution has long been recognized [1], [2], but its implications for evolutionary events such as speciation, extinction, and population structuring have only recently been verified and experimentally studied using molecular data and phylogenetic studies [3], [4], [5]. At multiple scales the patterns are clear and often surprising. For example, multiple marine taxa show strong molecular differentiation between populations in the Indian Ocean and Western Pacific, while little variation is present in the expansive Western Pacific [6]. Within the significantly smaller Southern California Bight, Hamm and Burton [7], using allozymes from *Haliotis* (abalone), found that genetic distance was independent of geographic distance over a 300 km sampling range, and suggested that limited larval dispersal is involved in generating genetic differentiation. Along the entire California coast, Moberg & Burton [8] have shown that sea urchins (*Strongylocentrotus*) also have very heterogeneous genetic compositions on both large and small geographic scales suggesting that, “the larval pool is not well mixed geographically (even on spatial scales <20 km), despite long planktonic larval duration.”

From these and other studies, a picture of the role of larval dispersal in contributing to the genetic makeup of populations is emerging, and it is one that often significantly differs [4], [5], [9], [10] from early expectations. This can be partly attributed to the fact that our understanding of the effects of larval dispersal remains incomplete due to (1) limited taxon sampling that has not included less abundant species with patchy distributions, and (2) the lack of attention given to the effects of ecological factors (e.g., habitat specificity and availability, predator-prey interactions, resource availability, etc.) on geographical distributions of genetic diversity.

This study examines the population structure of *Tectura paleacea* (Gastropoda; Patellogastropoda), a stenotopic species found only on its obligate seagrass host *Phyllospadix* [11], [12]. The distribution of *T. paleacea* is patchy on local and regional scales throughout the Northeastern Pacific (Vancouver, Canada to Baja California, Mexico [13]). This species is a broadcast spawner with lecithotrophic larvae that can spend anywhere from a few hours to a several days in the plankton [14], [15], [16], [17]. While some studies have shown that taxa with planktonic larvae tend to have low geographic population structure [18], [19], other studies find that long planktonic larval duration does not result in low geographic population structuring [8], [20]. These studies have tried to address the role of hydrogeography and fine scale physical oceanography in larval dispersal success, but conflicting results remain. Other studies have begun to examine the contribution of different ecological roles in population structuring. Ayre et al. [9] show that phylogeographic breaks within populations of marine organisms correspond to the degree to which the organism can utilize sheltered habitat, while Dawson [18] demonstrates a correlation between the distribution of edge-effect species and phylogeographic structure. Kelly & Eernisse [21] hypothesize that variability in near-shore sea surface temperature may be responsible for observed differences in the population structure of species with similar larval dispersal capabilities. These studies show a decoupling between larval dispersal strategies and phylogeographic patterns, but the contributions of specific local biotic factors to the differential success of larval dispersal remain untested.

The primary objective of this study was to measure the relative contribution of habitat specificity on larval dispersal and in turn biogeographic population structure within *T. paleacea* populations found along the Oregon-California coast. Specifically, we examine the role of seagrass in localized larval retention as an obstacle to gene flow. In doing so we determined the geographical distribution of mitochondrial lineages within *T. paleacea* and estimated genetic diversity. Furthermore, to evaluate the historical role of dispersal in structuring *T. paleacea* populations we compare modern-day morphological and mitochondrial geographic patterns.

## Materials and Methods

### Collections

**Figure 1:**
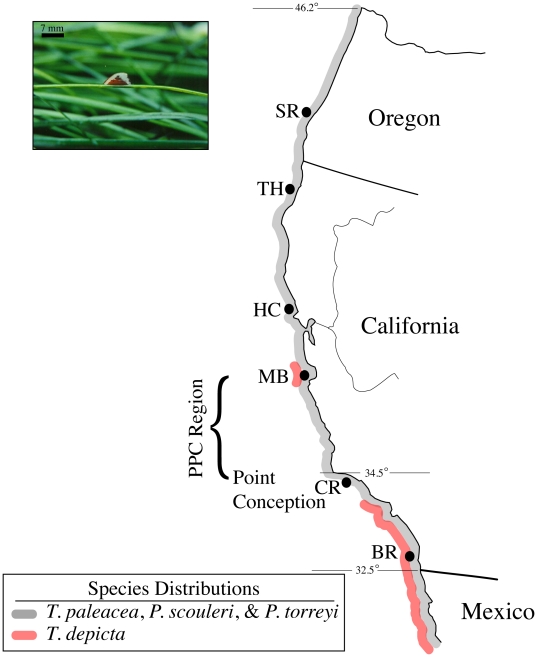
Map of six collection localities distributed along the California-Oregon coast. Locality abbreviations are as follows: SR - Simpson Reef, Cape Arago, OR; TH - Trinidad Head, Trinidad, CA; HC - Horseshoe Cove, Bodega, CA; MB - Monterey Bay, CA; CR - Coal Oil Point Reserve, Santa Barbara, CA; BR - Bird Rock, La Jolla, CA. Species distributions for *T. paleacea, T. depicta,* and *Phyllospadix* are indicated, as is the peri-Point Conception (PPC) region. The presence of *T. depicta* in MB appears to be a transient population that is introduced during El Niños (Zimmerman et al. 2001). The inset picture shows *T. paleacea* on a blade of *Phyllospadix* (scale bar is included).

Six localities with extensive *Phyllospadix* seagrass habitat were identified along the California-Oregon coast (see Figure 1). Each locality was approximately 2 degrees latitude apart and had *T. paleacea* populations present. The northern sampling boundary, determined by the northern range limit of *T. paleacea*, was Cape Arago, Oregon [22]. The southern sampling boundary was at Bird Rock in La Jolla, Caifornia, which is approximately 45 km north of the California-Mexico border.

Populations of *T. paleacea* were sampled from each locality at extreme low tides immediately after summer recruitment [14] during July 2002 and June 2003. Each sampling period entailed the identification of individual seagrass patches (defined as a continuous *Phyllospadix* rhizome covered substrate, separated by no less than one meter) within each locality. These patches were subsequently mapped using permanent markers embedded into surrounding boulders and triangulation techniques. All size classes of *T. paleacea* and their associated *Phyllospadix* blades were collected during timed searches from each *Phyllospadix* patch. Specimens of *T. paleacea* and blades of *Phyllospadix* were stored in 95% EtOH. Relative abundance was estimated in terms of *T. paleacea* individuals collected per person per unit search time. One-way ANOVA was used to test whether relative abundances for each locality were significantly different (ANOVA calculated using the statistical software package SPSS v11 [SPSS Inc., Chicago IL]).

A total of 763 individuals were collected and deposited in the University of California Museum of Paleontology (see Table S1 for accession numbers). For all subsequent analyses only adults were used (adults measure ≥3.5 mm in length [23]). Morphological measurements were taken from a sub-sample of 295 individuals and of these, 120 were used for sequencing. With permission, we also included sequence data from two individuals of *Tectura depicta* collected at San Diego, CA by Kristina D. Louie (unpublished).

### Sequencing

Small pieces of foot tissue were excised from a total of 120 *T. paleacea* adult individuals (10 individuals were sampled from each locality during the 2002 and again during the 2003 sampling period). Total genomic DNA was isolated using the “Animal Tissues” protocol included with the DNEasy Tissue Kit from QUIAGEN, Inc. Valencia CA. A 450 nucleotide fragment of cytochrome oxidase b mtDNA (Cytb) was amplified and sequenced using the following primers: cobF 5′ - GGW TAY GTW YTW CCW TGR GGW CAR AT - 3′; cobR 5′ - GCR TAW GCR AAW ARR AAR TAY CAY TCW GG - 3′ [24].

### Phylogenetic analysis

No length variation was observed between sequences allowing for manual alignment in Se-Al [25]. Cytb amino acid sequences were used to verify nucleotide sequences and alignment. All sequence types have been deposited in GenBank (see Table S1 for list of specimens and accessions numbers). Two additional Cytb sequence types from *T. depicta* (the sister taxon of *T. paleacea* [26]) were included with permission from Kristina D. Louie (unpublished) for outgroup comparisons and network rooting.

The maximum likelihood model that best fits the combined Cytb data was calculated using a hierarchical Akaike information criterion as implemented in modeltest v3.06 [27]. This test was chosen in place of the likelihood ratio test because it imposes a penalty for model complexity resulting in models with better predictive accuracy [28]. Haplotype relationships were reconstructed with the neighbor-joining (NJ) algorithm and Maximum Likelihood (ML) implementing the transversion model (TVM) in addition to the proportion of invariable sites (I) and gamma distribution (G) models describing variation among sites (TVM+I+G) model of evolution using PAUP* version 4.0b10 [29]. For ML analyses gaps were treated as missing data and the heuristic search algorithm was implemented for ten random addition sequence replicates. Support for haplotype relationships was calculated using fast bootstrap analysis (4168 replicates) [30], [31].

### Population genetics data analysis

Patterns of haplotype diversity were examined on regional scales by grouping individuals according to phylogenetic affinities. To further test if this grouping accurately reflects population connectivity, each population was serially allocated to unique blocks (i.e., all possible population groupings were examined) and AMOVA analyses [32], as implemented in arlequin v2.0 [33], were done on all partitions. Total molecular variance (σ^2^) was calculated as the sum of the covariance components F_ST_ (overall population variation), F_SC_ (within group variation), and F_CT_ (between group variation). Significance tests were based on 16,000 permutations.

Further population genetic data analyses were also carried out using arlequin v2.0. Haplotype and nucleotide diversity was estimated as a means to identify recent changes in effective population sizes. Tajima's [34] measure of nucleotide diversity (θ_π_) estimated from the mean number of pairwise nucleotide differences (π) was selected due to its incorporation of allele frequencies, making this statistic more biologically meaningful as compared to others [i.e., segregating sites (θ_S_)] [35]. Furthermore, the observed number of differences between haplotype pairs (i.e., mismatch distribution) was calculated in order to determine if populations are at demographic equilibrium. For example, while a bimodal mismatch distribution is indicative of demographic equilibrium, a unimodal distribution indicates that the population has most likely passed through a recent population expansion [36], [37].

### Morphological analyses

Length, width, and height measurements were taken from 25 *T. paleacea* individual collected from each locality during 2002 and again during 2003. A total of 50 individuals were measured from each locality except in the case of Cape Arago where only 20 individuals were collected in 2003 resulting in a total of 45 individuals being measured from this locality. In total, 295 individuals were measured for morphological analyses. *Phyllospadix* blade width was measured at the exact point from which each *T. paleacea* individual was removed from the blade. The relationship of these variables was examined across all localities using ANOVA and correlation statistics. Analyses were done with the statistical software package SPSS v11 (SPSS Inc., Chicago IL).

The relationship between locality and morphology was examined by comparing length:height ratios (LHR) of individuals from each locality. This measure has been established as an appropriate indicator of ecophenotypic variation in marine plant limpets previously [38]. Width was not considered in these comparisons due to the fact that individuals of *T. paleacea* spend their entire lives on *Phyllospadix* blades and stems. In turn, the width of these plant structures constrains *T. paleacea* shell width soon after individuals reach adult size therefore shell width is a reflection of ecophenotypic variation in the plant host and not in the associated limpet. The maximum width of 1.5–2.0 mm is reached when individuals are approximately 3.5 mm long [23]. In order to reduce the bias (i.e., inflating population differentiation) associated with the differential presence of juveniles at each locality (e.g., there were no juveniles found in SR and TH populations while juveniles were collected in abundance from more southern populations), all specimens collected that were smaller than 3.5 mm in length were removed from the morphological analysis.

The contribution of blade and stem constraints to the observed variation in LHR was tested using a series of three comparisons. (1) Shell width of *T. paleacea* was examined relative to each associated *Phyllospadix* blade (or stem) width across all localities. (2) ANOVA of *Phyllospadix* blade width was compared across all localities. In order to test if *Phyllospadix* blade width at any given locality was significantly affecting shell LHR, (3) the degree of ovalness, measured as width:length ratios (WLR), was compared across all localities.

## Results

### Phylogeography

**Figure 2:**
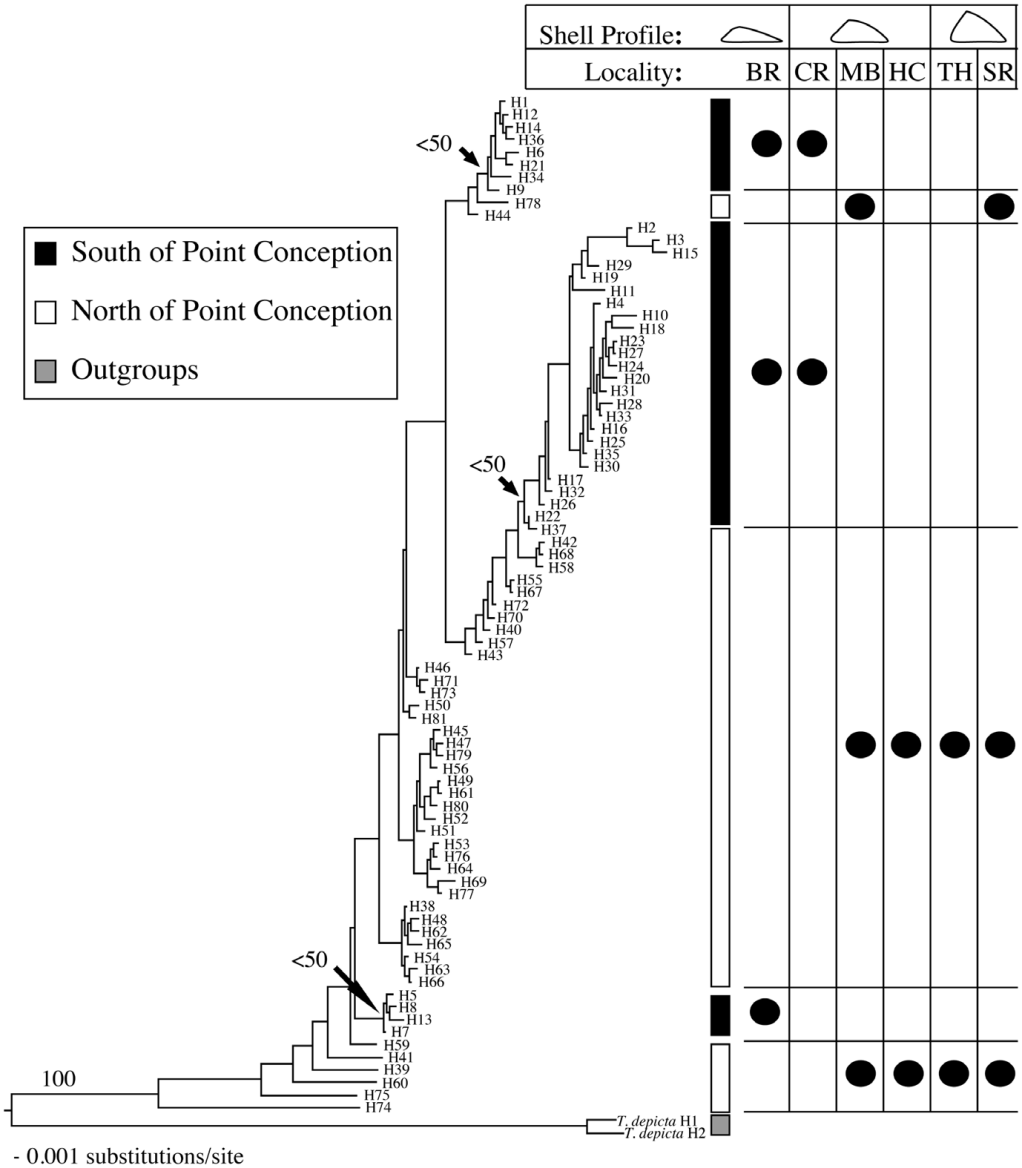
Neighbor-joining tree of partial Cytb mtDNA. Shows the relationship of 81 haplotypes and two outgroups haplotypes (*T. depicta* H1 & *T. depicta* H2). Corresponding haplotype localities are mapped, as is the average shell profile associated with each locality. Arrows indicate the ancestral node of southern clades nested within northern populations.

Of the 120 individuals sequenced, there were eighty-one unique haplotypes present. Twenty individuals were examined from each population resulting in the following haplotype distributions across the six sampled populations: 8 unique haplotypes from Simpson Reef, Cape Arago, OR (SR); 11 from Trinidad Head, Trinidad, CA (TH); 12 form Horseshoe Cove, Bodega, CA (HC); 13 from Monterey Bay, Monterey, CA (MB); 19 from Coal Oil Point Reserve, Santa Barbara, CA (CR); and 18 from Bird Rock, La Jolla, CA (BR). The haplotype relationships determined using a neighbor-joining algorithm are shown in Figure 2 with bootstrap values indicated above each branch of interest. The tree topology resulting from ML analysis is shown in Supplemental Figure S1 with bootstrap values indicated above each branch.

### Population genetics and demography

**Figure 3:**
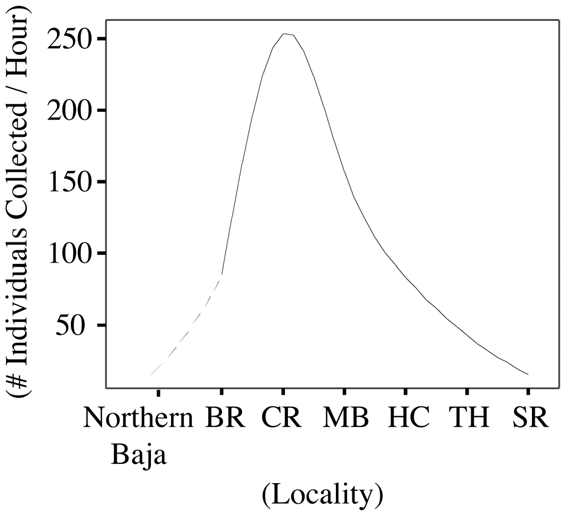
Relative abundance estimates (expressed in terms of *T. paleacea* individuals encountered per person per unit search time) plotted against all localities. The hatched line indicates the expected trend for population abundance in Baja, Mexico, the southern most limit of *T. paleacea* distribution.

The relative abundance of *T. paleacea* populations changed significantly across the sampled range. When plotted against localities by latitude, abundance data appear to have a normal distribution with the highest abundances at the center of the range of *T. paleacea* (Figure 3).

AMOVA analyses identified a strong geographic subdivision between the four populations sampled north of the peri-Point Conception (PPC) region and the two populations sampled south of the PPC region (see Table 1), with 32.94% of the total variance being attributed to population differences between these two regions. Only 1.59% of the total variance is attributed to differences between populations within these two regions. Fixation indices further support a reduction in gene flow across the PPC region with F_CT_ = 0.3294 indicating very great genetic differentiation [39]. These data collectively support a regional population subdivision between the populations sampled on either side of the PPC region.

**Table 1:** Two-region analysis of variance across six populations of *T. paleacea*.

Source of variation	Sum of squares	Variance components*	% of variation explained	Fixation index*	95% significance
Among regions	93.233	1.6564	32.94%	F_CT_ = 0.3294	*p≤0.00*
Among populations within each region	19.550	0.0798	1.59%	F_SC_ = 0.0237	*p≤0.00*
Within populations	375.300	3.2921	65.47%	F_ST_ = 0.3453	*p≤0.00*
*Total*	*488.083*	*5.02836*	*100%*		

AMOVA and phylogeographic pattern both indicate a geographical subdivision at the PPC region. The source of variation, percentage of variance explained, and fixation indices are included.

*Significance calculated at the 0.05 level.

**Figure 4:**
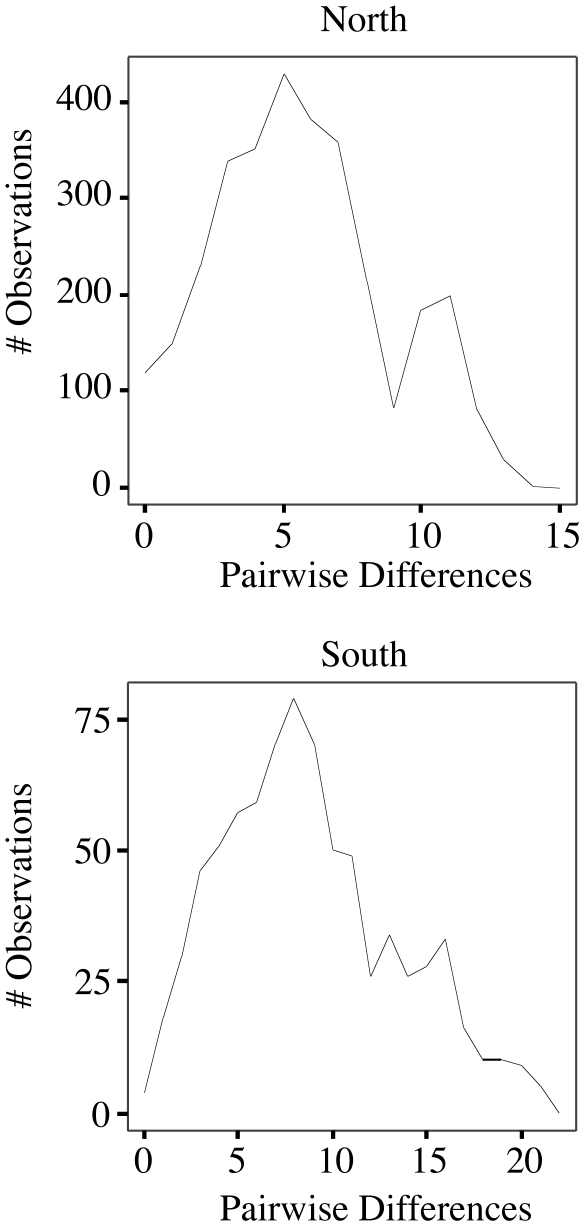
Mismatched distributions of pairwise base pair differences between Cytb haplotypes. Populations north of the PPC region have been at equilibrium longer than populations to the south with a smaller mean number of pairwise differences (π) in southern populations (π = 8.7731±4.1338) as compared to those in the north (π = 5.6652±2.7449). Southern populations show a relatively unimodal mismatch distribution indicative of more recent population expansion.

Mismatched distributions shown in Figure 4 indicate that populations north of the PPC region have been at equilibrium longer than populations in the south with a smaller mean number of pairwise differences (π) in northern populations (π = 5.6652±2.7449) as compared to those south of this geographic boundary (π = 8.7731±4.1338). While southern populations do not have an exact Poisson distribution, they do show a relatively unimodal mismatch distribution indicative of more recent population expansion [37]. These data are consistent with the observed decrease in genetic variation in northern populations. Haplotype diversity (H) and nucleotide diversity (θ_π_) are significantly smaller in northern population as compared to populations found south of the PPC region (Table 2).

**Table 2:** Genetic variation between populations north and south of the PPC region.

Population	Haplotype diversity (H)	Nucleotide diversity (θ_π_)	Mean # of pairwise differences
North of the PPC region	0.9620±0.0100	5.6652 (3.0417)	5.6652±2.7449
South of the PPC region	0.9949±0.0075	8.7731 (4.5922)	8.7731±4.1338

Standard deviation indicated in parentheses.

### Morphological patterns

**Figure 5:**
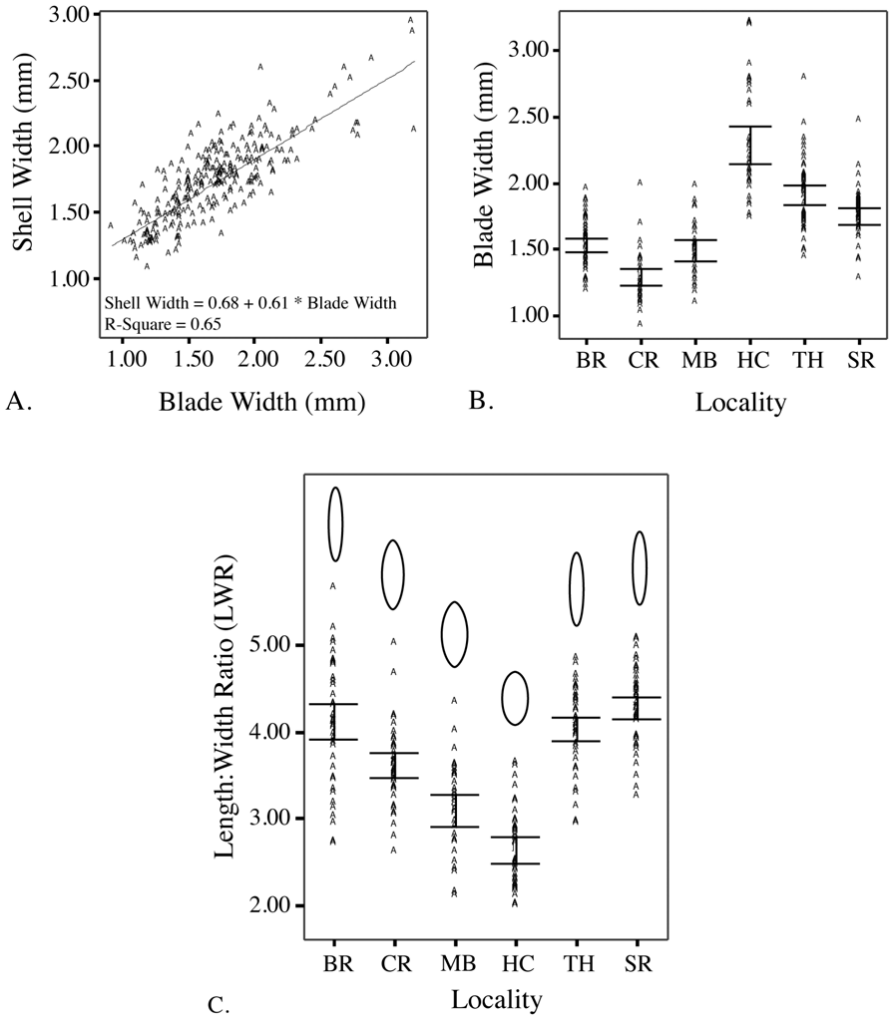
Morphological comparisons between *T. paleacea* and *Phyllospadix* . A) *T. paleacea* shell width is regressed against *Phyllospadix* blade (or stem) width across all localities resulting in a positive correlation. Scatter plots of B) *Phyllospadix* blade width and C) the degree of ovalness, measured as length:width ratios (LWR), compared across all localities are shown. Error bars show 95.0% confidence intervals. Note the lack of concordance between the patterns seen in B and C.

A positive correlation between blade width and shell width was observed (Figure 5a). ANOVA of *Phyllospadix* blade width compared across all localities resulted in significant differences (*p*<<0.05) between four groups of populations (Figure 5b). Average blade widths were largest in HC (mean = 2.2886) and smallest in CR (mean = 1.2930). ANOVA of length:width ratios (LWR) identified significant differences (*p*<<0.00) between four groups (BR + TH + SR; CR; MB; HC) of populations that did not correspond to the same four population groupings (BR + MB; CR; HC; TH + SR) found for blade width alone (see Figure 5b–c). The longest and most narrow forms were found in the SR population instead of the CR population, which had the narrowest average blade widths.

**Figure 6:**
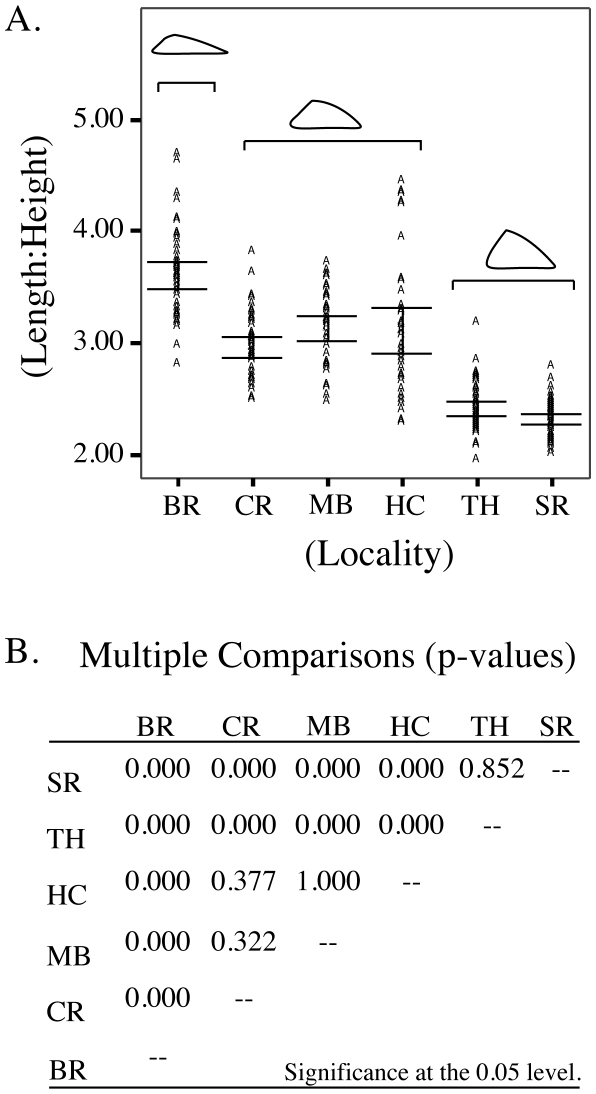
Differences between LHR across sampled populations. A) A scatter plot of LHR across all localities is shown. Error bars show 95.0% confidence intervals. B) ANOVA *p*-values are given for all comparisons. Significance is estimated at the 0.05 level using the Tukey post hoc test. A statistically significant break is evident between three groups of populations: 1) BR; 2) CR, MB, HC; and 3) TH, SR.

ANOVA and Tukey post hoc multiple comparisons of length:height ratios (LHR) across all localities indicated a statistically significant break between three groups (BR; CR + MB + HC; TH + SR) of populations (Figure 6a–b). BR had the largest LHR (*p*<<0.00) indicating that overall shell shape is characterized by elongated shell length and low apex height. The opposite was true for TH and SR populations, which had significantly smaller LHR (*p*<<0.00) indicating that shell shape is shorter in length with a relatively high apex. The three populations found between the most northern and southern extremes (CR, MB, and HC) were statistically indistinguishable form each other (*p* = 0.206), and were characterized by an intermediate shell shape.

## Discussion

### Historic and phylogeographic patterns

A diverse patellogastropod fauna found in Tertiary fossil deposits from the Dominican Republic include species that were closely associated with marine plants [40]. These records indicate the presence of marine seagrass limpets in the Caribbean by the late Miocene. The first record of a *T. paleacea-*like marine plant limpet in the subtropical Pacific comes from a Pleistocene deposit in the Sea of Cortez (Lindberg unpublished). As seagrass limpets expanded their range into the temperate North Pacific during the late Pliocene and early Pleistocene, populations in the subtropical Pacific and Caribbean went extinct [40]. These fossil data indicate that the ancestral populations of both *T. paleacea* and *T. depicta,* the sister taxon of *T. paleacea* [26], originated in the Caribbean and moved through the Isthmus of Panama sometime before the closure of the isthmus 3.1–3.5 million years ago [41], [42] and subsequently expanded their range into northern latitudes. The extant range of *T. depicta* is restricted from northern Baja to central California reflecting ancestral distributions.

AMOVA results indicate that a barrier to gene flow between northern and southern populations of *T. paleacea* exists somewhere between Santa Barbara and Monterey (i.e., the PPC region). While this region encompasses the well-known biogeographic boundary of Point Conception [43], [44], [45], this reduction in gene flow is not necessarily associated with Point Conception itself; more sampling would need to be done across the PPC region to confirm the exact location of the geographic barrier to gene flow [46]. Historical patterns examined in association with AMOVA results indicate that northern population isolation resulted from a range expansion rather than a selective sweep. Evidence supporting a recent range expansion includes lower nucleotide diversity estimates north of Santa Barbara, the southern distribution of *T. depicta*, and concordant patterns seen in a variety of taxa that share a similar coastal distribution across the Northeastern Pacific [47], [48], [49].

From a phylogenetic perspective, haplotype relationships predicted from NJ and ML analyses further support the presence of a partial barrier to gene flow in the PPC region. As expected, due to the low levels of genetic variation present between our sampled populations, the tree resulting from ML analysis has less resolution than the NJ tree and neither have significant support values (Figure 2 & Figure S1). Nonetheless NJ and ML patterns of haplotype relationships indicate that an ancestral northern assemblage of populations has given rise to multiple clusters of extant southern populations independently. The presence of three (potentially more if ML results are considered) reciprocally monophyletic clades of haplotypes originating south of the PPC region nested within the paraphyletic clade of haplotypes originating north of the PPC region is indicative of at least three independent dispersal events originating from northern populations followed by rapid coalescence giving rise to the extant southern populations (Figure 2). In light of the known historical data that supports a range shift from the north to the south this pattern fits well with the fact that there is a persistent eddy extending from spring through summer south of Point Conception, which functionally makes Point Conception a one-way biogeographical boundary where larvae can only disperse north to south during the majority of the reproductive season for most marine species in this region [50], [51]. Therefore, these flow patterns combined with more recent historical temperature shifts, which may have facilitated regional population contractions/expansions, could explain this pattern of repeated north to south dispersal. Given the available data we cannot rule out the possibility that *T. paleacea* populations may have gone extinct entirely south of Point Conception during the late Pliocene and early Pleistocene. If this was the case then these dispersal events represent range expansion into areas where *T. paleacea* had previously gone extinct. This raises the possibility that southern populations are being maintained as sink populations deriving from northern larval source populations. This scenario would have significant impact on conservation strategies being implemented in rocky intertidal environments along the California-Oregon coast.

### Mechanisms regulating population structure

It has been generally accepted that seagrass canopies alter the hydrodynamic conditions of the space they occupy [52]. Flume tank observations and field studies have demonstrated that seagrass beds reduce flow [53], [54] and dampen vertical wave energy [55], [56]. Recent evidence supports the long-standing hypothesis that this reduction in water flow acts as a mechanism to increases particle trapping [57], [53]. The expectation is that the observed increase in particle trapping within seagrass beds should have significant effects on larval retention and subsequent patterns of recruitment for broadcast spawning taxa associated with seagrasses.

Given these findings from previous studies, the obligate association of *T. paleacea* with *Phyllospadix* would suggest increased levels of population structure due to decreased flow environments and subsequently increased larval retention within *Phyllospadix* beds. While we did not directly measure the level of larval dispersal ability associated with any given seagrass bed, our data indicate that the presence of any larval retention due to attenuated flow in seagrass beds is not enough to act as a population structuring force for populations of *T. paleacea* distributed along the California-Oregon coast. The lack of collection site-specific population structure indicates high levels of gene flow between sites. Given the lack of dispersal ability by adults, the possibility of this gene flow being a result of adult dispersal as opposed to larval dispersal is unlikely. While adult dispersal potential may exist via individuals hitchhiking on drifting clumps of seagrass, this has yet to be documented.

The data presented here lend support to the hypothesis that a biogeographic boundary within the PPC region is acting as the primary mechanism driving genetic population structuring in *T. paleacea*. This result is consistent with Avise's [58] hypothesis that concordance exists between biogeographic and intraspecific phylogeographic boundaries. This indicates a local decoupling of reproduction from larval recruitment [59] and in turn argues in support of pre-settlement larval availability as the primary population structuring force acting within these populations. These findings have important implications for the ecological importance of open-coast seagrass communities on larval recruitment. Specifically, these findings suggest that open-coast seagrass communities act primarily as larval sources and do not actively facilitate increased larval settlement.

### Phylogeography and morphology

There is a clear decoupling between patterns of morphological variation and population structure. Shape change is shown in Figure 5c and indicates a shift from a low profile, elongate form in the south to a shorter and taller profile in the north. While there are three statistically significant clusters of morphotypes distributed across the sampled distribution of *T. paleacea*, the boundaries between these clusters do not correspond to the PPC region, the biogeographic boundary associated with the phylogeographic break between northern and southern populations (Figure 1). Furthermore, variability seen in *Phyllospadix* blade morphology (previous studies have also shown that *Phyllospadix* morphology systematically varies across its range [60]) cannot account for the morphological differences seen in *T. paleacea* populations given the lack of overlap between three of the four population groupings identified by ANOVA for LWR and blade width across localities (Figure 5b–c).

Two possible mechanisms might explain the pattern of morphological variation observed across the sampled distribution of *T. paleacea*. The first is natural selection followed by local adaptation, while the second is ecophenotypic plasticity in response to local environmental variability. If natural selection was acting to produce the morphological variation observed between populations, given the fact that Cytb is among the most rapidly evolving markers found within the mitochondrial genome of gastropods [42], we would expect to see a concordance between molecular and morphological patterns. Instead the presence of incomplete lineage sorting suggests that natural selection is not acting on the populations within each morphologically unique region, although the possibility remains that there has not been enough time for mitochondrial genes to reflect selective pressure [61].

The second possible mechanism is differential ecophenotypic expression as a response to habitat heterogeneity. A number of studies have identified similar morphological trends associated with local ecophenotypic responses to variable environments [62], [63], [64]. Although currently there are few ecological data available for *T. paleacea*, making it difficult to determine the extent of ecophenotypic plasticity, studies on other intertidal snails have demonstrated that flow intensity can significantly alter foot size and shell morphology [65], [66], [67] as can predation intensity [65], [68], [69] and food supply [70]. All of these factors may be contributing to the region-specific morphological variation observed in *T. paleacea* populations. Although there are limited food supply and predation data available to begin to address this question, transplant experiments could be used to test for plasticity and in turn determine the necessity for further ecological and/or molecular data.

Morphological variation within *Phyllospadix* may contribute to the differences observed between *T. paleacea* populations. Three morphological species of *Phyllospadix* (*P. serrulatus, P. scouleri,* and *P. torreyi)* occur along the coast of North America. Individuals of *T. paleacea* feed exclusively on the leaf epidermis of *P. scouleri* [71] and occasionally on *P. torreyi* [72]. This feeding strategy has been attributed to the availability of proteins and absence of phenolic substances in blade epidermal cells of *Phyllospadix* [73]. While studies have indicated strong support for the differentiation of *P. serrulatus* from *P. scouleri* and *P. torreyi*, few data support the separate species designation of *P. scouleri* and *P. torreyi* [60], [74]. Additionally, intermediate forms of these two species have been identified [75] suggesting that hybridization may occur. However, comparison data do exist for leaf epidermal differences between these three named species [73], suggesting that epidermal cell shape and size differ between *P. scouleri* and *P. torreyi*. These data, combined with data that indicate latitudinal variability in *P. scouleri* and *P. torreyi* [60], may be associated with the morphological variation seen in *T. paleacea* populations living on these seagrasses.

*Phyllospadix* beds provide a partial defense against many larger benthic predators due to the inaccessibility of thin floating blades where *T. paleacea* is found. However, smaller predators, such as the sea star *Leptasterias hexactis*, are commonly found in these beds [76]. Laboratory experiments conducted by Fishlyn and Phillips [76] demonstrated that *T. paleacea* individuals show no escape response as a result of contact with *L. hexactis* individuals. Instead, they suggest that *T. paleacea* is chemically camouflaged. If *T. paleacea* uses chemical camouflage as its primary defense against predators, it is unlikely that predation intensity could explain the region-specific variability seen in shell morphology. If we are to identify the mechanisms regulating ecophenotypic plasticity within *T. paleacea*, detailed studies examining the ecological role of *T. paleacea* in seagrass habitats and its ecophenotypic plasticity potential are needed.

### Summary

This study identifies population structure of *T. paleacea* that is concordant with the biogeographic barrier at the PPC region in California. Historical data suggest that extant populations originated in southern subtropical regions and have made their way across this barrier only since the late Pliocene or early Pleistocene. Although larval dispersal was not directly measured, the pattern of population structuring observed indicates that seagrass communities, which are known to alter flow regimes within their environment, do not affect larval dispersal ability enough to impact on the connectivity of *T. paleacea* populations. Furthermore, morphological patterns are not concordant with the molecular patterns observed. These findings support the role of local habitat variability in maintaining differential ecophenotypic expression across the distribution of *T. paleacea*, suggesting that highly localized conservation efforts may not be as effective as large-scale conservation efforts in near shore marine environments with diverse life history components.

## Supporting Information

Figure S1 The resulting strict consensus tree topology using ML analysis. ML analysis resulted in two trees with the likelihood score 1542.74406 and few nodes with significant bootstrap support. Bootstrap values (4,168 replicates) are indicated above each branch. Southern clades are highlighted in red while northern clades are in black.
(TIF)

Table S1 (DOC)
