# Supplementary figures and images for: Genetic Population Structure of Tectura paleacea: Implications for the Mechanisms Regulating Population Structure in Patchy Coastal Habitats
Source: PLoS One. 2011 Apr 7;6(4):e18408. doi: 10.1371/journal.pone.0018408 (PMC3072387; doi:10.1371/journal.pone.0018408)

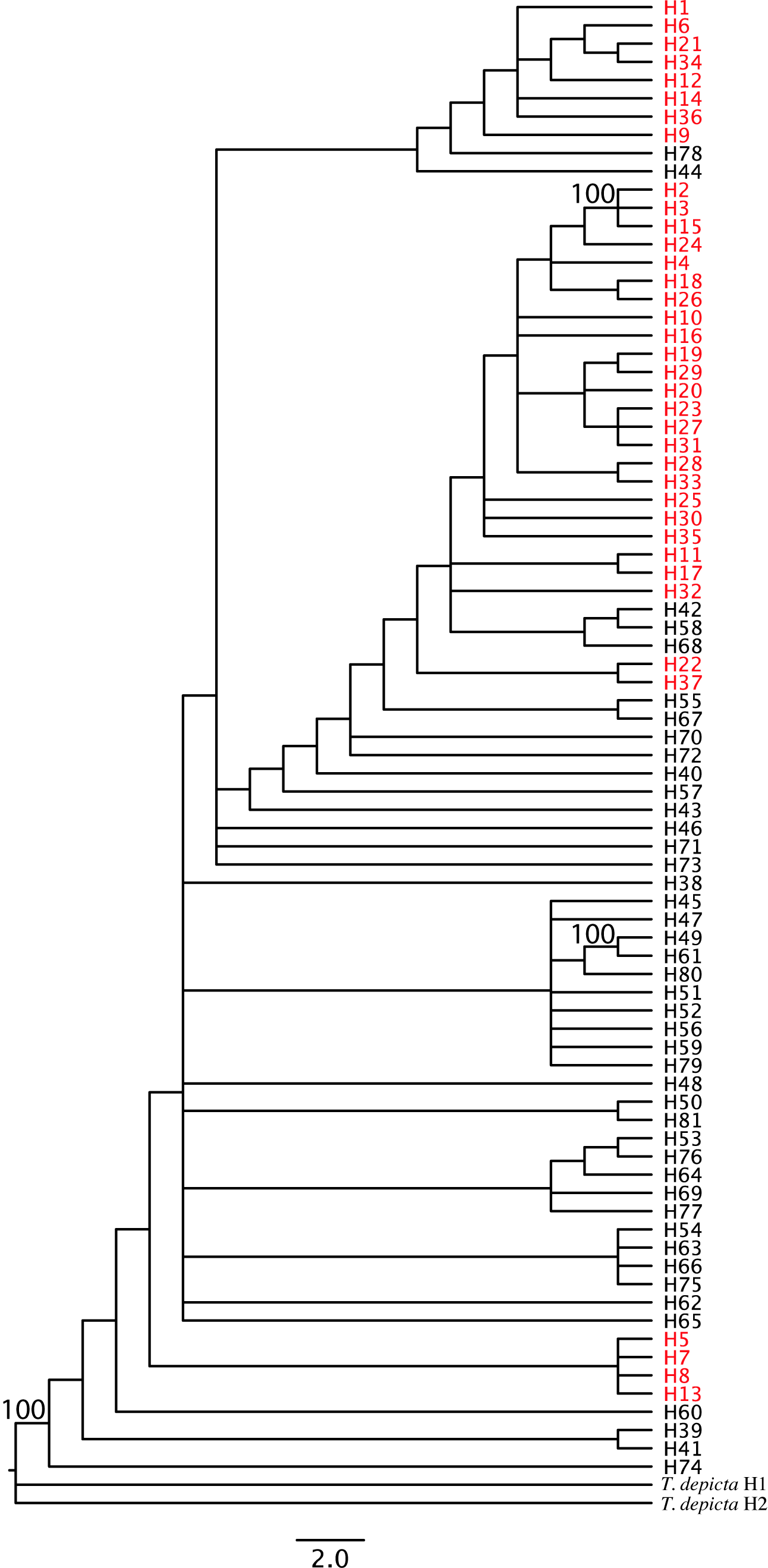

Supplement: Figure S1 — The resulting strict consensus tree topology using ML analysis. ML analysis resulted in two trees with the likelihood score 1542.74406 and few nodes with significant bootstrap support. Bootstrap values (4,168 replicates) are indicated above each branch. Southern clades are highlighted in red while northern clades are in black. (TIF) [file pone.0018408.s001.tif]
