# Supplementary material for: Genetic Population Structure of Tectura paleacea: Implications for the Mechanisms Regulating Population Structure in Patchy Coastal Habitats
Source: PLoS One. 2011 Apr 7;6(4):e18408. doi: 10.1371/journal.pone.0018408 (PMC3072387; doi:10.1371/journal.pone.0018408)
Supplement: Table S1 — (DOC) [file pone.0018408.s002.doc]

**Table S1** Haplotype data including locality information, UCMP specimen numbers and Genbank accession numbers (JF523205-JF523324). Collection year indicated for each specimen at each locality. Locality identifications correspond to those shown in Fig. 1 of main text.

| Locality | **2002** | | | **2003** | | |
| --- | --- | --- | --- | --- | --- | --- |
|  | **UCMP Specimen Accession #** | **GenBank Accession #** | **Haplotype** | **UCMP Specimen Accession #** | **GenBank Accession #** | **Haplotype** |
| **SR** | 38663 | JF523306 | H38 | 38723 | JF523319 | H42 |
|  | 38664 | JF523310 | H42 | 38724 | JF523320 | H42 |
|  | 38665 | JF523312 | H49 | 38725 | JF523323 | H49 |
|  | 38666 | JF523307 | H49 | 38726 | JF523322 | H52 |
|  | 38667 | JF523313 | H72 | 38727 | JF523315 | H76 |
|  | 38668 | JF523311 | H72 | 38728 | JF523316 | H77 |
|  | 38669 | JF523308 | H72 | 38729 | JF523317 | H78 |
|  | 38670 | JF523305 | H72 | 38730 | JF523318 | H79 |
|  | 38671 | JF523309 | H74 | 38731 | JF523321 | H80 |
|  | 38672 | JF523314 | H75 | 38732 | JF523324 | H81 |
| **TH** | 38673 | JF523288 | H42 | 38733 | JF523304 | H41 |
|  | 38674 | JF523287 | H46 | 38734 | JF523303 | H49 |
|  | 38675 | JF523289 | H49 | 38735 | JF523301 | H49 |
|  | 38676 | JF523285 | H63 | 38736 | JF523298 | H49 |
|  | 38677 | JF523286 | H64 | 38737 | JF523296 | H49 |
|  | 38678 | JF523290 | H65 | 38738 | JF523297 | H60 |
|  | 38679 | JF523291 | H66 | 38739 | JF523295 | H70 |
|  | 38680 | JF523292 | H67 | 38740 | JF523299 | H71 |
|  | 38681 | JF523293 | H68 | 38741 | JF523300 | H72 |
|  | 38682 | JF523294 | H69 | 38742 | JF523302 | H73 |
| **HC** | 38683 | JF523273 | H39 | 38743 | JF523283 | H46 |
|  | 38684 | JF523270 | H42 | 38744 | JF523280 | H46 |
|  | 38685 | JF523265 | H42 | 38745 | JF523279 | H46 |
|  | 38686 | JF523266 | H51 | 38746 | JF523275 | H49 |
|  | 38687 | JF523269 | H52 | 38747 | JF523276 | H57 |
|  | 38688 | JF523267 | H52 | 38748 | JF523277 | H58 |
|  | 38689 | JF523268 | H53 | 38749 | JF523278 | H59 |
|  | 38690 | JF523271 | H54 | 38750 | JF523281 | H60 |
|  | 38691 | JF523272 | H55 | 38751 | JF523282 | H61 |
|  | 38692 | JF523274 | H56 | 38752 | JF523284 | H62 |
| **MB** | 38693 | JF523249 | H38 | 38753 | JF523255 | H38 |
|  | 38694 | JF523245 | H38 | 38754 | JF523264 | H39 |
|  | 38695 | JF523246 | H39 | 38755 | JF523257 | H42 |
|  | 38696 | JF523247 | H40 | 38756 | JF523262 | H46 |
|  | 38697 | JF523250 | H41 | 38757 | JF523260 | H46 |
|  | 38698 | JF523248 | H41 | 38758 | JF523256 | H46 |
|  | 38699 | JF523251 | H42 | 38759 | JF523258 | H47 |
|  | 38700 | JF523252 | H43 | 38760 | JF523259 | H48 |
|  | 38701 | JF523253 | H44 | 38761 | JF523261 | H49 |
|  | 38702 | JF523254 | H45 | 38762 | JF523263 | H50 |
| **CR** | 38703 | JF523234 | H28 | 38763 | JF523244 | H37 |
|  | 38704 | JF523233 | H27 | 38764 | JF523243 | H36 |
|  | 38705 | JF523232 | H26 | 38765 | JF523241 | H35 |
|  | 38706 | JF523231 | H25 | 38766 | JF523240 | H34 |
|  | 38707 | JF523230 | H24 | 38767 | JF523239 | H33 |
|  | 38708 | JF523229 | H23 | 38768 | JF523238 | H32 |
|  | 38709 | JF523228 | H22 | 38769 | JF523237 | H31 |
|  | 38710 | JF523227 | H21 | 38770 | JF523236 | H30 |
|  | 38711 | JF523226 | H20 | 38771 | JF523235 | H29 |
|  | 38712 | JF523225 | H19 | 38772 | JF523242 | H7 |
| **BR** | 38713 | JF523214 | H10 | 38773 | JF523224 | H18 |
|  | 38714 | JF523213 | H9 | 38774 | JF523223 | H17 |
|  | 38715 | JF523212 | H8 | 38775 | JF523222 | H16 |
|  | 38716 | JF523211 | H7 | 38776 | JF523220 | H15 |
|  | 38717 | JF523210 | H6 | 38777 | JF523218 | H14 |
|  | 38718 | JF523209 | H5 | 38778 | JF523217 | H13 |
|  | 38719 | JF523208 | H4 | 38779 | JF523216 | H12 |
|  | 38720 | JF523207 | H3 | 38780 | JF523215 | H11 |
|  | 38721 | JF523206 | H2 | 38781 | JF523219 | H7 |
|  | 38722 | JF523205 | H1 | 38782 | JF523221 | H3 |
